# Microwave Roasting as an Alternative to Convection Roasting: Sensory Analysis and Physical Characterization of Dark Chocolate

**DOI:** 10.3390/foods12040887

**Published:** 2023-02-19

**Authors:** Joachim J. Schouteten, Valérie Lemarcq, Davy Van de Walle, Eleni Sioriki, Koen Dewettinck

**Affiliations:** 1 Department of Agricultural Economics, Ghent University, Coupure Links 653, 9000 Gent, Belgium; 2 Department of Food Technology, Safety and Health, Food Structure & Function Research Group (FSF), Faculty of Bioscience Engineering, Ghent University, Coupure Links 653, 9000 Gent, Belgium

**Keywords:** roasting, trained panel, consumer, microwave, preference, flavor, cocoa beans, chocolate

## Abstract

Roasting cocoa beans by means of microwave radiations seems to be a potential alternative to convection roasting, but little is known about the impact of this method on the perceived flavor profile of the chocolate. Therefore, this research focused on revealing the flavor perception of chocolate produced with microwave roasted cocoa beans assessed by both a trained panel and chocolate consumers. Samples of 70% dark chocolate produced from cocoa beans microwave roasted at 600 W for 35 min were compared with samples of 70% dark chocolate produced from cocoa beans convectively roasted at 130 °C for 30 min. Non-significant differences (*p* > 0.05) in the measured physical properties, such as color, hardness, melting, and flow behavior, showed that chocolate produced from microwave roasted cocoa beans can exhibit the same physical qualities as convection roasted chocolate. Moreover, combined discriminative triangle tests, with 27 judgements in total, performed by a trained panel, showed that each type of chocolate exhibited distinctive characteristics (d’-value = 1.62). Regarding the perceived flavor, “cocoa aroma” was cited as significantly higher for the chocolate produced from microwave roasted cocoa beans (*n* = 112) compared to chocolate produced from convection roasted cocoa beans (*n* = 100) by consumers. Both preference and willingness to buy were higher, though insignificant at a 5% level, for the microwave roasted chocolate. A final potential benefit (studied in this research) of microwave roasting cocoa beans is the reduced energy consumption, which was estimated at 75%. Taking all these results together, the microwave roasting of cocoa is shown to be a promising alternative to convection roasting.

## 1. Introduction

The roasting of beans of the *Theobroma cacao* [1] variety is an essential processing step in the production of chocolate. Traditionally, cocoa beans, or cocoa nibs, are roasted by means of convection and/or conduction. This heat treatment, which is typically applied at a temperature range from 110 to 160 °C for approximately half an hour, is responsible for the moisture content reduction and the development of the typical cocoa flavor due to the Maillard reaction taking place during roasting, leading to the formation of compounds such as methylpyrazines and Strecker aldehydes [2,3,4]. However, research has shown that this easily applicable process is accompanied by several drawbacks. Specifically, the heat transfer from outside towards the inside of the beans can result in a burnt surface, whereas the center might be less affected. Hence, a non-homogenous bean heating occurs, which could result in potentially enhanced bitterness. In addition, the heating efficiency within the beans is rather low. This phenomenon is typically observed in high fat products, such as cocoa beans, because of their poor rate of heat transfer [5,6]. Furthermore, this roasting process leads to the transfer of a respectable amount of cocoa butter to the outer shell, which is economically disadvantageous [7].

To overcome these disadvantages, several alternatives were proposed. One of the promising substitutes for the conventional roasting methods is microwave roasting, as it is believed to be more energy-efficient [5]. By applying electromagnetic radiation in the form of microwaves, which have frequencies between 300 MHz and 300 GHz, heat is created in the entire bean via ionic polarization and dipole rotation [8]. The suitability of this technique to roast cocoa beans mainly relies on the high fat content of this product. The low specific heat of fats results in a relatively low heat requirement to increase the temperature of the product [9,10]. Despite the auspicious character of microwave roasting, the available literature regarding this topic is rather scarce. Specifically, no research was found to investigate the effect on the sensory perception of chocolate produced from cocoa beans roasted via this alternative technique.

The goal of this explorative research is to study the flavor perception of chocolate produced from microwave roasted cocoa beans. A trained panel and chocolate consumers were both used to perform a sensory analysis of the chocolates in order to obtain insights regarding the perceived flavor profile by means of analytical and affective tests. In addition, physical properties, such as dry matter, color, hardness, melting, and viscosity, were measured to investigate if chocolate produced from microwave roasted cocoa beans could be of comparable quality to chocolate made from convectively roasted beans.

## 2. Materials and Methods

### 2.1. Coco Bean Roasting

Two promising treatments were selected based upon results from an earlier study which compared multiple treatments varying in time/power/temperature [7]. Both chocolates were produced from fermented and dried cocoa beans (origin: Ecuador, variety: mixed) provided by Cargill (Schiphol, The Netherlands). The first type of chocolate was produced from cocoa beans that were convectively roasted in batches of 2 kg for 30 min at 130 °C in a drum roaster (Roaster 102, Selmi Group, Santa Vittoria d’Alba, Italy) and reached a dry matter content of 98% *w*/*w*. The second type of chocolate was produced from cocoa beans that were roasted in batches of 900 g in a domestic microwave (MC32K7085KT, Samsung, Seoul, Republic of Korea) for 35 min at 600 W, and in this way, a similar, non-significant (*p* > 0.05) dry matter content (98 % *w*/*w*) was obtained.

### 2.2. Chocolate Production

Roasted cocoa beans were winnowed (Winn-15 Mini Winnower, Cacao Cucina^®^, Bottom Line Process Technologies, Inc., Clearwater, FL, USA) and, subsequently, the cocoa nibs were subjected to a coarse grinding (UMC5 Stephan mixer, Stephan bvba, Nazareth, Belgium) and a fine grinding step (W-1-S ball mill, Royal Duyvis Wiener, Koog aan de Zaan, The Netherlands) to produce cocoa liquor. The cocoa liquors were then used to produce 70% cocoa dark chocolate on a 5 kg scale, with the following composition: 30% sugar, 64.65% cocoa liquor, 5% cocoa butter (CB), and 0.35% soy lecithin. The chocolate production process consisted of mixing, refining, conching, tempering, and molding steps. 

#### 2.2.1. Mixing 

In the mixing step, pre-broken sugar (Barry Callebaut, Wieze, Belgium) and cocoa liquor were blended in a VEMA BM 30/20 planetary mixer (Machinery Verhoest NV/Vema Construct, Izegem, Belgium) at 45 °C for 20 min, with a fat content of up to 22% *w*/*w*.

#### 2.2.2. Refining

The resulting dough-like paste was fed onto the Exakt 80S three-roll refiner (Exakt Apparatebau GmbH & Co., KG, Norderstedt, Germany) with a roll distance setting of 2-1. Refining was performed at 35 °C and 400 rpm.

#### 2.2.3. Conching

A Buhler Elk’Olino conche (Richard Frisse GmbH, Bad Salzuen, Germany) device was used to carry out the conching process. During the dry conching step, the refined flakes were conched at 55 °C and 1200 rpm in the clockwise direction (mixing phase) for 2 h and then at 80 °C and 1200 rpm in the counterclockwise direction (shearing phase) for 4 h, and both steps were performed with an open lid. During this process, the consistency of the mass was periodically observed. If it was perceived as too dry by a skilled operator, extra cocoa butter (Belcolade, Erembodegem, Belgium) was added to improve mixing. Indicators of a too dry mass are a powdery consistency, persisting distinct balls, or the attachment of lumps on the mixing element [11]. If necessary, the addition was performed in small increments to prevent it from becoming too fluid, as this may hinder the evaporation of moisture and undesired volatile acids. The added cocoa butter was considered in the following wet conching phase to assure that the amount of total fat in the system was consistent in all four productions. As for the wet conching stage, the rest of the ingredients were added as follows: pre-conched cocoa liquor (i.e., cocoa liquor not added during mixing but separately conched at 80 °C for 4 h and 1200 rpm in the counterclockwise direction), the remaining cocoa butter, and 0.35% lecithin (Soya International Ltd., Cheshire, UK). The liquefaction of the chocolate samples was performed with a closed lid twice for 15 min at 45 °C and 2400 rpm, whereby a mixing step was followed by a shearing step. 

#### 2.2.4. Tempering and (De)Molding

The resulting chocolate samples were tempered manually on a marble table. The tempered chocolates were molded into square-shaped tablets (33 mm × 33 mm × 3 mm) and were cooled in a thermostatic cabinet for at least 1 h at 12 °C. This cooling process was necessary to promote further crystallization of the βV crystals in order to facilitate demolding. The next day, aluminum foil was used to wrap the chocolates to prevent quality changes during storage. The chocolates were matured for at least 2 weeks in a thermostatic cabinet at 15 °C before further analyses.

### 2.3. Sensory Evaluation

#### 2.3.1. Trained Panel

The trained panel used in this study was established in 2017. Recruitment and intensive trainings were carried out, as described in the manuscript of Rottiers [12]. Participants provided informed consent and the study complied with the principles established by the Declaration of Helsinki and in respect of the EU General Data Protection Regulation (GDPR). The study was approved by the Ethics Committee of Ghent University Hospital (B670201835745). A discriminative triangle test [13] was conducted using the EyeQuestion software (Logic8 B.V., Elst, The Netherlands) by the trained panel to investigate whether the chocolates were distinguishable. This test was performed in triplicate and performed once by 18, once by 17, and once by 16 panel members. The varying number of total panel members performing these sensory tests is related to certain assessors experiencing health-related issues at the time of tasting (due to the COVID-19 pandemic). All samples of the triangle tests were delivered to the testers’ homes, and at-home tests were conducted, when an undisturbed environment could be guaranteed. The samples were coded using random 3-digit numbers and presented following a Williams Latin square design [14].

#### 2.3.2. Consumer Panel

##### Recruitment

Potential tasters were recruited via e-mail and selected when they fulfilled the selection criteria (e.g., absence of nut allergy). In total, 130 persons participated the consumer test, with a gender distribution of 60/40 (women/men). All participants were dark chocolate consumers, living in Belgium, and older than the age of 18, with an overall mean age of 33 years old. Participants provided informed consent, and the study complied with the principles established by the Declaration of Helsinki and the EU General Data Protection Regulation (GDPR) [15]. The chocolates were home-delivered, and data were collected with EyeQuestion software (Logic8 B.V., Elst, The Netherlands). The samples, identified with a 3-digit random number, were presented following a Williams Latin square design [16]. Anonymous collection of the data was guaranteed, and it involved no sensitive data. Participants were not reimbursed for their participation.

##### Consumer Test

Check-all-that-apply tests (CATA) were used to gain information about the characteristics of the chocolate aroma and flavor [17]. This easy technique avoids scaling problems often encountered by untrained people, as there is no demand to rate the intensity [18,19]. Moreover, previous studies showed that there was a high correlation between the citation frequency and intensity of a certain descriptor [20]. Consumers had to check off the boxes that were applicable to the aroma and flavor profile of the specific chocolate. Both aroma and flavor attributes were selected based on results of a roundtable discussion including six experts with an extensive knowledge of describing aromas and flavors present in chocolate products. The selected terms to describe the aroma were: roasted, spicy, fruity, cocoa, floral, smoky, and burnt. The selected terms for describing flavor were: roasted, spicy, fruity, cocoa, floral, bitter, buttery, acid, sweet, dry, caramel, and orange. In addition to the CATA test, consumers were asked to indicate their preference and willingness to buy.

### 2.4. Physical Properties

#### 2.4.1. Dry Matter

The dry matter content (% *w*/*w*) of the roasted cocoa beans and the produced chocolates was determined via oven-drying, according the official method AOAC 931.04 [21]. The dry matter content of the chocolates was measured in triplicate.

#### 2.4.2. Color

The color of the two chocolate samples is expressed according to the CIELAB color space. L*, a*, and b* values were measured by a Minolta CM-2500d spectrophotometer (Konica Minolta Sensing Osaka, Tokyo, Japan) in triplicate. The applied settings were as follows: 10° observation angle, D65 light source, with the specular component excluded. In addition, the browning index (BI), an indicator for the brown color of the chocolates, was calculated from the obtained L*, a*, and b* values, according the equation reported in ZZaMan and Yang [22]:

$$BI=\frac{\left[100\;\left(x-0.31\right)\right]}{0.17}\;\;\;\;\;\;\;\;\;\;\;\;\;\;\;\;\;\;\;with\;x=\frac{\left(a^{*}+1.75L^{*}\right)}{\left(5.64L^{*}+a^{*}-3.012b^{*}\right)}$$

In addition, the Δ*E* value, representing the difference in color between the two samples, was measured according to the following equation, found in Żyżelewicz, et al. [23]:

$$\Delta E=\sqrt{{\left(\;L_{1}^{*}-L_{2}^{*}\right)}^{2}+{\left(a_{1}^{*}-a_{2}^{*}\;\right)}^{2}+{\left(b_{1}^{*}-b_{2}^{*}\;\right)}^{2}\;}$$

#### 2.4.3. Hardness

The hardness (N) of the chocolate tablets was analyzed in triplicate via a needle penetration test performed on a 5942 Instron Texture Analyzer (Norwood, MA, USA) equipped with a 500 N load cell and Bluehill^®^ software (v4.03). The penetration test was initiated when the needle felt a threshold of 0.2 N upon touching the chocolate tablets and continued until a penetration depth of 5 mm at a rate of 2 mm/s.

#### 2.4.4. Melting Profile

The melting profile of the chocolates was monitored via differential scanning calorimetry (Q1000 DSC, TA instruments, New Castle, DE, USA), as mentioned in the work of Tran et al. [24]. Each measurement was performed in triplicate, and the measured melting peaks were described by their onset, maximum, offset temperature (°C), and melting enthalpy (J/g).

#### 2.4.5. Apparent Viscosity

The AR2000ex rheometer (TA Instruments, New Castle, DE, USA) was used to assess the flow behavior of the chocolates in triplicate, using the concentric cylinder system (conical bob; stator inner radius of 15 mm and rotor outer diameter of 14 mm; gap of 5920 µm). The applied method was based on the International Confectionery Association (ICA) 46 method, as described in the work of Saputro et al. [25]. The apparent viscosity (Pa.s) of both chocolates was reported for increasing shear rates ranging from 2 s^−1^ to 50 s^−1^.

### 2.5. Estimated Energy Consumption

The estimated energy consumption (kJ/kg) of both roasting treatments was calculated based on the electrical power requirement (J/s) of both devices, the roasting time, and the batch size, as presented in following equation:

$$\mathrm{Energy}\;\mathrm{consumption}\;\left(\frac{\mathrm{kJ}}{\mathrm{kg}}\right)=\frac{\mathrm{Electrical}\;\mathrm{power}\;\mathrm{requirement}\;\left(\frac{\mathrm{kJ}}{s}\right)\times \mathrm{Roasting}\;\mathrm{time}\;\left(s\right)}{\mathrm{Batch}\;\mathrm{size}\;\left(\mathrm{kg}\right)}$$

### 2.6. Statistical Analysis

All tests were performed at a significance level of 5%. Results of the triangle tests were statistically analyzed via both binomial tests and Thurstonian modeling, resulting in *p*-values and d’-values, respectively. Whereas XLSTAT 2020 (Microsoft^®^, Redmond, DC, USA) was used to calculate the d’-values, EyeOpenR 5.0.6.5. software (Logic8 B.V., Elst, The Netherlands) was used to calculate the binomial *p*-values. Other outcomes of the consumer tests were statistically compared in SPSS 27 (IBM, New York, NY, USA) using different types of tests. Whereas check-all-that-apply and willingness to buy tests were statistically evaluated via Mc-Nemar tests, preference was analyzed via as a simple paired preference test following the methods of Lawless and Heymann [13]. The physical properties of the chocolates were also statistically compared in SPSS 27 (IBM, New York, NY, USA). Prior analysis via a paired sample t-test, the normality of these data and the equality of variances were checked via QQ-plots and Levene’s tests, respectively.

## 3. Results and Discussion

### 3.1. Trained Panel

#### Triangle Tests

In total, 51 triangle tests were performed during three different sessions (T1: *n* = 18, T2: *n* = 17, T3: *n* = 16). As shown in Table 1, both the binominal *p*-value, as well as the d’-value derived from Thurstonian modeling, are displayed for the individual sessions, as well as for the total number of assessments. A *p*-value below 0.05 and a d’-value above 1.5 are both indicators of significant differences between the products [26]. According to Kunert and Meyners [27], the results of the three triangle test sessions could be combined, even if these are replicates. In this way, a higher power to detect differences between products is obtained. The results showed that for two out of three triangle tests, both chocolates could be distinguished by the panel members. Only triangle test T2 resulted in a *p*-value above 0.05 and a d’-value below 1.5. Moreover, by combining all data (T1 + T2 + T3), an additional confirmation was given that chocolate produced from microwave and convectively roasted cocoa beans could be distinguished at a 5% significance level.

### 3.2. Consumer Test

#### 3.2.1. Check-All-That-Apply Aroma and Flavor Attributes

**Figure 1:**
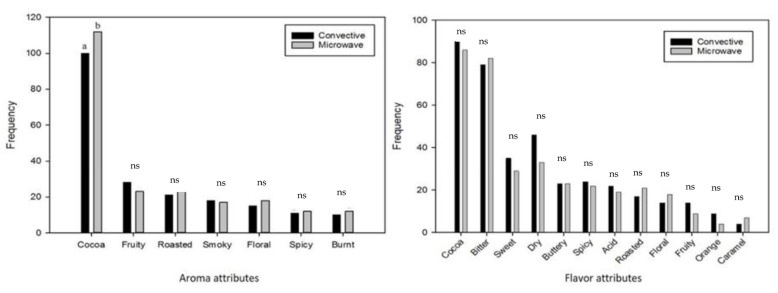
Bar chart representing the usage frequencies of 7 aroma attributes (**left**) and 12 flavor attributes (**right**) detected by consumers (*n* = 130) in chocolate produced from convectively and microwave roasted cocoa beans. Only cocoa aroma was significantly more applicable for the microwave chocolate samples, as indicated by different lower case letters a and b; the others were non-significant (ns) (*p* < 0.05).

The citation frequency of the aroma attributes, as presented by the left bar chart in Figure 1, shows that a comparable aroma profile could be expected from both chocolates. An exception was observed for the predominant aroma, cocoa, which was cited as significantly higher for the chocolate produced from microwave roasted cocoa beans (*n* = 112) compared to the convectively roasted cocoa beans (*n* = 100). Research of Lemarcq, Monterde, Tuenter, Van de Walle, Pieters, Sioriki, and Dewettinck [7] found higher concentrations of 2-methylbutanal, which are associated with a more intense cocoa aroma [28], when chocolates were produced from microwave roasted beans compared to convectively roasted beans.

Regarding flavor perception by mouth, no significant differences (*p* > 0.05) were detected between the CATA results of the chocolates (Figure 1). For both chocolates, cocoa and bitterness flavors were the most cited, suggesting that these were the dominant flavor attributes. This might not come as a surprise when serving consumers a 70% dark chocolate sample. The bitter flavor might have been especially overwhelming, thus masking the more subtle differences in the delicate fruity and floral flavor notes.

#### 3.2.2. Willingness to Buy and Preference 

**Figure 2:**
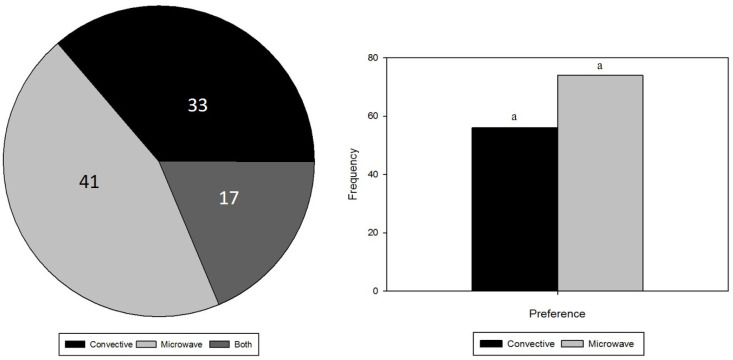
Willingness to buy (**left, *n* = 94**) and preference (**right, *n* = 130**) regarding chocolate produced from convectively and microwave roasted beans (in number of persons). Regarding willingness to buy, only 94 participants (out of 130) indicated that they were willing to buy at least one sample. No significant differences (ns) were detected for either the willingness to buy or the preference between the chocolates based on a 5% significance level.

Figure 2 visualizes a similar tendency for the results of the two tests used to the check the affectivity of consumers towards the two products. Both willingness to buy and preference suggest that the chocolate produced from microwave roasted cocoa beans was more appreciated by the consumers. Nevertheless, neither parameter was significantly different using a 95% confidence interval. Although, a significant difference (*p* < 0.05) in preference would be obtained if only 3 persons (*n* = 77) switched their preferences to chocolate made from microwave roasted cocoa beans.

### 3.3. Physical Properties

The physical properties of the produced chocolates were examined through the analyses of the dry matter content, color, hardness, melting behavior, and apparent viscosity. The results of these experiments, with the exception of apparent viscosity, can be found in Table 2.

**Figure 3:**
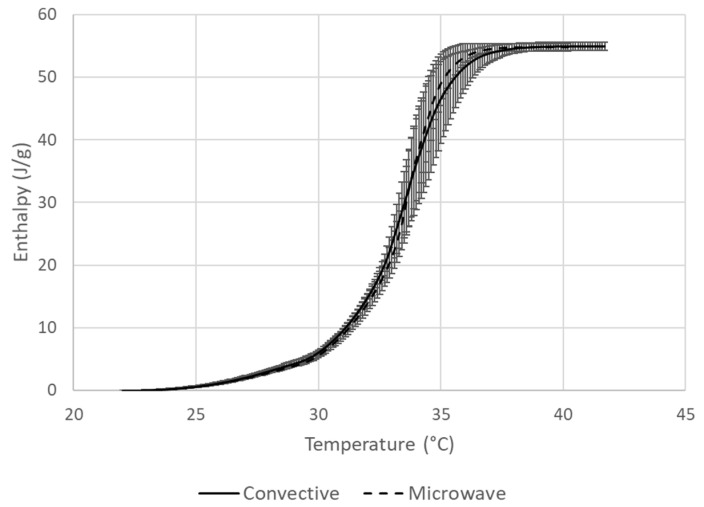
Melting enthalpy profile of chocolates produced from convective roasted cocoa beans and microwave roasted cocoa beans using differential scanning calorimetry (*n* = 3).

No significant differences (*p* > 0.05) were detected between the dry matter (DM) of the chocolates. The DM levels were above 99.5% *w*/*w*, indicating that after the roasting process, supplementary evaporation occurred, especially during the dry conching step. These high DM levels should ensure the avoidance of aggregation and lump formation [29]. With regard to color, no difference between the chocolate samples was observed, as no significant differences (*p* > 0.05) were detected for L*, a*, and b* values. The calculated browning index representing the intensity of brown color for each chocolate, was also judged as similar. These results were confirmed by the calculated ∆E value of below 1, indicating that the color difference is not obvious when observed by the human eye [23]. In terms of hardness, no significant differences (*p* > 0.05) were detected between the hardness of the two chocolate samples. With respect to melting behavior, comparable onset, offset, and maximum temperatures (°C) of the melting peaks were found for both chocolates. Moreover, the heat required to melt each chocolate, as measured by the melting enthalpy (J/g), was not significantly higher (*p* > 0.05) for either of the samples, implying that both chocolates exhibited a similar melting behavior and induced a similar cooling sensation. The melting enthalpy profile of the cholates can be found in Figure 3.

**Figure 4:**
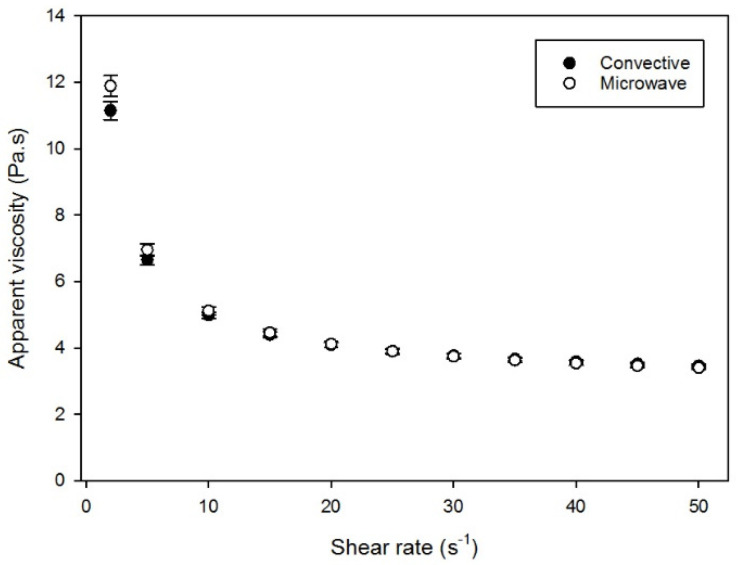
Viscosity (Pa.s) measured as a function of applied shear rate (s^−1^) to compare the flow behavior of chocolate produced from convectively (●) or microwave (○) roasted cocoa beans.

The flow properties were also studied, as it is known that flavor perception depends on the order and rate of contact, which is related to the viscosity of chocolate melted in the mouth [30]. As can be seen from the graph presented in Figure 4, comparable apparent viscosities were measured at varying shear rates for both chocolates. Only for a very low shear rate of 2s^−1^ was a significant difference (*p* < 0.05) in apparent viscosity measured. However, as can be seen from the table reported in the work of Mezger [31], processes such as the chewing and swallowing of a food product are represented by shear rates between 10 to 100s^−1^; therefore, the relevance of this measuring point is relatively limited. The flow behavior data has been can be found in Figure S1 and the flow parameters (fitted with the appropriate rheological model) is depicted in Table S1. Additional rheological measurement with a thinner film in the range of μm and nm, as suggested by Stokes et al. [32], would provide supplementary information to study tribology components such as lubrication, in addition to the typical astringent mouthfeel when consuming dark chocolate.

### 3.4. Estimated Energy Consumption

All parameter values used to estimate energy consumption are displayed in Table 3. For this small scale roasting experiment, it could be concluded that the microwave roasting treatment was more energy efficient. This increased energy efficiency does not result from a shorter roasting time, nor a higher bean capacity of the equipment, but because of the lower energy consuming equipment that was used in this study.

## 4. Conclusions

This study confirms the hypothesis that microwave roasting could be a suitable alternative to the convection roasting of cocoa beans. In addition to the lower energy consumption detected on a small scale, chocolate with desirable sensory properties could be produced from microwave roasted cocoa beans. Moreover, a significantly higher number of consumers associated the chocolate produced from microwave roasted cocoa beans with displaying a stronger cocoa aroma. The larger number of consumers willing to buy, and their higher preference for, this type of chocolate, although both insignificant (*p* > 0.05), illustrates the more than acceptable sensory and physical properties of chocolate produced from microwave roasted cocoa beans. The acceptable sensory quality was also confirmed by the very similar physical properties measured for both types of chocolates.

On a small scale, the applicability of using microwaves to roast cocoa beans is proven. This technique can be used, for example, by local cocoa farmers to explore the flavor profile of their cocoa beans by producing chocolate at a lower investment and operational cost. 

Notwithstanding these promising results, further investigations are necessary. With respect to the applicability of microwave roasting cocoa beans on an industrial scale, further research is required. When upscaling, other types of equipment will be necessary, as well as the selection of a different set of parameter values. By varying the time-power input combinations, the varying roasting degrees will affect the final flavor profile of the produced chocolate. To fully understand the impact of time and power input on the flavor profile of chocolate produced from microwave roasted cocoa beans, it is highly recommended to perform kinetic modeling studies. Further research should also examine the impact of the roasting technique on the nutritional and chemical composition of chocolate, e.g., polyphenols and antioxidants. In addition, a sensory evaluation of chocolates produced from cocoa beans roasted at different time-power combinations is highly supported in order to further map their corresponding flavor profiles.

Finally, the economic aspect, partly determined by energy efficiency, will also be an important decisive factor in determining whether this technique is a worthy alternative for the chocolate industry.

## Figures and Tables

**Table 1:** Correct and total results of triangle tests (including statistical *p*- and d’-values) for the 70% dark chocolate samples produced from convectively roasted (convectively: 130 °C–30 min) and microwave roasted (microwave: 600 W–35 min) cocoa beans performed in three different sessions (T1 = first session, T2 = second session, T3 = third session).

Triangle Test	Correct	Incorrect	Total	*p*-Value	d’-Value
T1	11 ^a^	7 ^b^	18	0.014	2.032
T2	6 ^a^	11 ^a^	17	0.522	0.466
T3	10 ^a^	6 ^b^	16	0.016	2.103
T1 + T2 + T3	27 ^a^	24 ^b^	51	0.003	1.618

Note: ^a b^ superscripts indicate significant differences detected between the number of correct and incorrect answers on the triangle tests.

**Table 2:** Dry matter content, color (L, a*, b*, BI, ∆E), hardness, and melting (Tm onset, max, offset, and melting enthalpy) parameters measured for chocolate produced from convectively and microwave roasted beans.

Physical Properties		Convection	Microwave
**Dry Matter Content (% *w*/*w*)**		99.730 ^a^ ± 0.024	99.65 ^a^ ± 0.13
Color			
	L	24.93 ^a^ ± 0.37	25.83 ^a^ ± 0.22
	a*	6.71 ^a^ ± 0.13	6.67 ^a^ ± 0.10
	b*	4.47 ^a^ ± 0.15	4.16 ^a^ ± 0.20
	Browning index (BI)	38.9 ^a^ ± 1.3	35.9 ^a^ ± 1.3
	Δ*E*	0.95	
Hardness (N)		10.720 ^a^ ± 0.066	10.58 ^a^ ± 0.11
Melting parameters			
	T_m, onset_ (°C)	24.86 ^a^ ± 0.30	24.78 ^a^ ± 0.25
	T_m, max_ (°C)	34.16 ^a^ ± 0.74	34.00 ^a^ ± 0.55
	T_m, offset_ (°C)	37.1 ^a^ ± 1.2	36.56 ^a^ ± 0.50
	Melting enthalpy (J/g)	54.97 ^a^ ± 0.65	54.78 ^a^ ± 0.59

Note: All measurements were performed in triplicate (*n* = 3), and the results are expressed by the mean ± standard deviation (X ± SD). ^a^ The same superscript indicates that no significant differences were detected between the chocolates, based on a 5% significance level (*p* > 0.05).

**Table 3:** Comparison of electrical energy consumption between the Selmi roaster (convection: 130 °C–30 min) and the Samsung domestic microwave oven (microwave: 600 W–35 min), taking into account the applied roasting conditions used in this study.

Equipment	Electrical Power Requirement (kJ/s)	Roasting Duration (s)	Maximum Bean Capacity (kg)	Energy Consumed during Roasting (kJ/kg)
Convection	15	1800	2	13,500
Microwave	1.4	2100	0.9	3266

## Data Availability

The data are not publicly available, as participants did not provide consent to share their data.

## Supplementary Materials

The following supporting information can be downloaded at: https://www.mdpi.com/article/10.3390/foods12040887/s1, Figure S1: Shear stress as a function of increasing shear rates for chocolates produced from convective and microwave roasted cocoa beans (*n* = 3).; Table S1: Flow parameters (mean ± S.D.) of the chocolates produced from convectively and microwave roasted cocoa beans (*n* = 3).

## References

[1] Jean-Marie E., Jiang W., Bereau D., Robinson J.-C. (2022). Theobroma cacao and Theobroma grandiflorum: Botany, Composition and Pharmacological Activities of Pods and Seeds. Foods.

[2] Aprotosoaie A.C., Luca S.V., Miron A. (2016). Flavor chemistry of cocoa and cocoa products—An overview. Compr. Rev. Food Sci. Food Saf..

[3] Noor A.F., Budi T.S.A. (2019). Roasting Equipment for Cocoa Processing. Drying and Roasting of Cocoa and Coffee.

[4] Kruszewski B., Obiedziński M.W. (2020). Impact of raw materials and production processes on furan and acrylamide contents in dark chocolate. J. Agric. Food Chem..

[5] Krysiak W. (2011). Effects of convective and microwave roasting on the physicochemical properties of cocoa beans and cocoa butter extracted from this material. Grasas Y Aceites.

[6] Ryynänen S. (2002). Microwave Heating Uniformity of Multicomponent Prepared Foods. Ph.D. Thesis.

[7] Lemarcq V., Monterde V., Tuenter E., Van de Walle D., Pieters L., Sioriki E., Dewettinck K. (2022). Flavor diversification of dark chocolate produced through microwave roasting of cocoa beans. LWT.

[8] Singh R.P., Heldman D.R., Singh R.P., Heldman D.R. (2014). Introduction to Food Engineering.

[9] Teferra T.F. (2019). Engineering properties of food materials. Handbook of Farm, Dairy and Food Machinery Engineering.

[10] Hebbar H.U., Rastogi N.K. (2012). Microwave heating of fluid foods. Novel Thermal and Non-Thermal Technologies for Fluid Foods.

[11] Beckett S.T., Fowler M.S., Ziegler G.R. (2017). Beckett’s Industrial Chocolate Manufacture and Use.

[12] Rottiers H. (2019). From Genetics to Flavor Dynamics and Sensory Profiling of Fine and Bulk Ecuadorian Cocoa. Ph.D. Thesis.

[13] Lawless H.T., Heymann H. (2010). Sensory Evaluation of Food: Principles and Practices.

[14] Wakeling I.N., MacFie H.J.H. (1995). Designing consumer trials balanced for first and higher orders of carry-over effect when only a subset of k samples from t may be tested. Food Qual. Prefer..

[15] EU General Data Protection Regulation (GDPR) Regulation (EU) 2016/679 of the European Parliament and of the Council of 27 April 2016 on the Protection of Natural Persons with Regard to the Processing of Personal Data and on the Free Movement of Such Data, and Repealing Directive 95/46/EC (General Data Protection Regulation). In OJ 2016 L 119/1; 2016. https://eur-lex.europa.eu/eli/reg/2016/679/oj.

[16] MacFie H.J., Bratchell N., Greenhoff K., Vallis L.V. (1989). Designs to balance the effect of order of presentation and first-order carry-over effects in hall tests. J. Sens. Stud..

[17] Wang B., Shen C., Zhao T., Zhai X., Ding M., Dai L., Gai S., Liu D. (2022). Development of a Check-All-That-Apply (CATA) Ballot and Machine Learning for Generation Z Consumers for Innovative Traditional Food. Foods.

[18] Varela P., Ares G. (2012). Sensory profiling, the blurred line between sensory and consumer science. A review of novel methods for product characterization. Food Res. Int..

[19] Marques C., Correia E., Dinis L.-T., Vilela A. (2022). An overview of sensory characterization techniques: From classical descriptive analysis to the emergence of novel profiling methods. Foods.

[20] Jaeger S.R., Chheang S.L., Jin D., Roigard C.M., Ares G. (2020). Check-all-that-apply (CATA) questions: Sensory term citation frequency reflects rated term intensity and applicability. Food Qual. Prefer..

[21] Flanyak J. (1995). Cacao bean and its products. AOAC Off. Methods Anal..

[22] ZZaMan W., Yang T.A. (2013). Effect of superheated steam and convection roasting on changes in physical properties of cocoa bean (*Theobroma cacao*). Food Sci. Technol. Res..

[23] Żyżelewicz D., Krysiak W., Nebesny E., Budryn G. (2014). Application of various methods for determination of the color of cocoa beans roasted under variable process parameters. Eur. Food Res. Technol..

[24] Tran P.D., Van Durme J., Van de Walle D., De Winne A., Delbaere C., De Clercq N., Phan T.T.Q., Phuc Nguyen C.H., Tran D.N., Dewettinck K. (2016). Quality attributes of dark chocolate produced from Vietnamese cocoa liquors. J. Food Qual..

[25] Saputro A.D., Van de Walle D., Caiquo B.A., Hinneh M., Kluczykoff M., Dewettinck K. (2019). Rheological behaviour and microstructural properties of dark chocolate produced by combination of a ball mill and a liquefier device as small scale chocolate production system. LWT.

[26] Nishida M., Lestringant P., Cantu A., Heymann H. (2021). Comparing classical descriptive analysis with modified descriptive analysis, modified rate-all-that-apply, and modified check-all-that-apply. J. Sens. Stud..

[27] Kunert J., Meyners M. (1999). On the triangle test with replications. Food Qual. Prefer..

[28] Afoakwa E.O., Paterson A., Fowler M., Ryan A. (2008). Flavor formation and character in cocoa and chocolate: A critical review. Crit. Rev. Food Sci. Nutr..

[29] Afoakwa E.O., Paterson A., Fowler M. (2007). Factors influencing rheological and textural qualities in chocolate—A review. Trends Food Sci. Technol..

[30] Beckett S.T. (2019). The Science of Chocolate.

[31] Mezger T. (2020). The Rheology Handbook: For Users of Rotational and Oscillatory Rheometers.

[32] Stokes J.R., Boehm M.W., Baier S.K. (2013). Oral processing, texture and mouthfeel: From rheology to tribology and beyond. Curr. Opin. Colloid Interface Sci..

