# Supplementary material for: Microwave Roasting as an Alternative to Convection Roasting: Sensory Analysis and Physical Characterization of Dark Chocolate
Source: Foods. 2023 Feb 19;12(4):887. doi: 10.3390/foods12040887 (PMC9957408; doi:10.3390/foods12040887)
Supplement: Supplementary file 1 [file foods-12-00887-s001.zip › foods-2131562-supplementary.pdf]

Supplementary materials

To accompany

Microwave Roasting as an Alternative to Convection Roasting: Sensory Analysis and Physical Characterization of Dark Chocolate

Joachim J. Schouteten[1, *], Valérie Lemarcq[2], Davy Van de Walle[2], Eleni Sioriki[2] and Koen Dewettinck[2]

[1] Department of Agricultural Economics, Ghent University, Coupure Links 653, Gent 9000, Belgium
[2] Department of Food Technology, Safety and Health, Food Structure & Function Research Group (FSF), Faculty of Bioscience Engineering, Ghent University, Belgium

* Correspondence: Joachim.Schouteten@ugent.be; Tel.: +3292645945

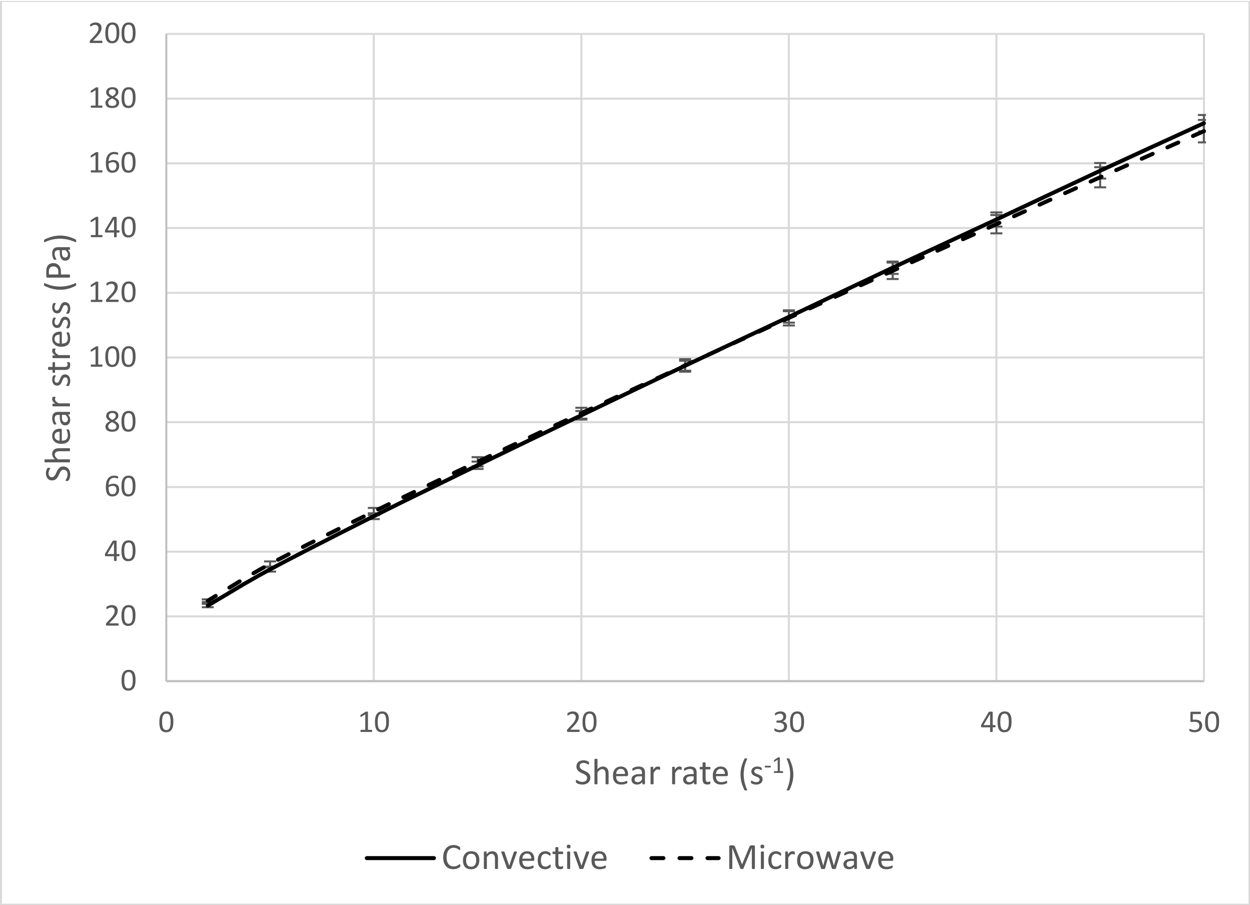

Figure S1. Shear stress in function of increasing shear rates for chocolates produced from convective and microwave roasted cocoa beans (n=3).

	Convection	Microwave
σCA, downward (Pa)	6,43±0,18	7,73 ± 0,13
ηCA, downward (Pa.s)	2200±23	2056±30
Thixotropy (Pa)	1,20±0,32	1,34±0,33
η* @ 50 s^{-1} (mPa.s)	3458±39	3400±49

Table S1. Flow parameters (mean ± S.D.) of the chocolates produced from convective and roasted cocoa beans (n=3). No significant differences were found for the different flow parameters ($p<0.05$).
